# Full-length transcriptome characterization and comparative analysis of *Gleditsia sinensis*

**DOI:** 10.1186/s12864-023-09843-y

**Published:** 2023-12-08

**Authors:** Feng Xiao, Yang Zhao, Xiurong Wang, Xueyan Jian

**Affiliations:** 1 Institute for Forest Resources and Environment of Guizhou, Key Laboratory of Forest Cultivation in Plateau Mountain of Guizhou Province, College of Forestry, Guizhou University, Guiyang, 550025 Guizhou China; 2 School of Continuing Education, Yanbian University, Yanji, 133002 Jilin China

**Keywords:** *Gleditsia sinensis*, Comparative transcriptome, PacBio SMRT, Ka/Ks

## Abstract

As an economically important tree, *Gleditsia sinensis* Lam. is widely planted. A lack of background genetic information on *G. sinensis* hinders molecular breeding. Based on PacBio single-molecule real-time (SMRT) sequencing and analysis of *G. sinensis*, a total of 95,183 non-redundant transcript sequences were obtained, of which 93,668 contained complete open reading frames (ORFs), 2,858 were long non-coding RNAs (LncRNAs) and 18,855 alternative splicing (AS) events were identified. Genes orthologous to different *Gleditsia* species pairs were identified, stress-related genes had been positively selected during the evolution. AGA, AGG, and CCA were identified as the universal optimal codon in the genus of *Gleditsia*. *EIF5A* was selected as a suitable fluorescent quantitative reference gene. 315 Cytochrome P450 monooxygenases (*CYP450s*) and 147 uridine diphosphate (UDP)-glycosyltransferases (*UGTs*) were recognized through the PacBio SMRT transcriptome. Randomized selection of *GsIAA14* for cloning verified the reliability of the PacBio SMRT transcriptome assembly sequence. In conclusion, the research data lay the foundation for further analysis of the evolutionary mechanism and molecular breeding of *Gleditsia*.

**Supplementary Information:**

The online version contains supplementary material available at 10.1186/s12864-023-09843-y.

## Background

Plants in the genus *Gleditsia*, have been used as local and traditional medicines in many regions, especially in China [1]. In China, there are six *Gleditsia* species and two varieties:* G. sinensis*, *G. australis*, *G. fera*, *G. japonica*, *G. microphylla*, *Gleditsia japonica* var. *delavayi*, *Gleditsia japonica *var. *velutina*, and the introduced species *G. triacanthos* [2]. *G. sinensis* (Fam.: *Leguminosae*; Subfam.: *Caesalpinioideae*), a deciduous tree or shrub, is a diploid species with 2n = 28 chromosomes [3, 4], resistant to drought, cold, and pollution, highly stress-resistant, and one of the first colonizing tree species as farmland turned into forest [5]. The branches are grayish to deep brown; thorn robust, terete, conical, often branched; leaves alternate, often clustered, one or two times even-pinnately compound; flowers are polygamous; seeds one to many, ovoid or elliptical, flat or sub-cylindrical, fruiting 5–12 months of the year [6].

The main economic features of *G. sinensis* involve three parts: pods, seeds, and thorns, which are widely used in the pharmaceutical industry because of their extremely high medicinal value [7, 8]. *G. sinensis* seeds are rich in pectin and protein components, used as thickeners, stabilizers, binders, gelling agents, etc*. G. sinensis* seed is an unconventional source for industrial gum with structure and properties similar to guar gum. As a medicinal plant, the economic value of *G. sinensis* is becoming increasingly important, with *G. sinensis* planted in rural farms more commonly for spines and pods in many countries, benefiting farmers’ incomes. However, there are many problems, such as inconsistent varieties, poor management techniques, low saponin and seed yields, poor quality, and low yield in actual production. Currently, there is a lack of complete background genetic information on *G. sinensis*. The first second-generation (RNA-seq) transcriptome of a *G. sinensis* mixed sample was sequenced and reported in 2014 [9], and RNA-Seq quickly became the technology of choice for gene-expression profiling. Wu et al. [10] analyzed green, purple, and yellow *G. sinensis* leaves, and found that differences in the expression of pigment-related genes were related to leaf color. The RNA-seq sequencing and analysis of different developmental tissues and stages of *G. sinensis* thorns were performed [11]. The stem tip of different species within the *Gleditsia* genus were sequencing using the RNA-seq [4]. Third-generation Pacific BioSciences (PacBio) single-molecule real-time (SMRT) sequencing does not need to interrupt RNA fragments, and direct reverse transcription can be used to obtain full-length cDNA, which can generate long reads of up to 60 kb [12–14]. Pacbio SMRT sequencing is widely used to obtain long reads and assemble a high quality reference transcriptome. PacBio-SMRT sequencing can provide a high-quality reference transcriptome for non-genomic species. In *Pinus massoniana,* 41,407 isoforms with an average length of 1822 bp were obtained through PacBio SMRT sequencing [15]. In order to obtain comprehensive genetic information of *G. sinensis*, various tissues were collected for PacBio SMRT sequencing to obtain the background transcriptome. Subsequently, positive selection pressure analysis was performed on the genes, analyzing codon usage bias in the *Gleditsia* genus, and stable reference genes with consistent expression were selected.

## Results

### Quality assessment and composition of raw data

**Fig. 1:**
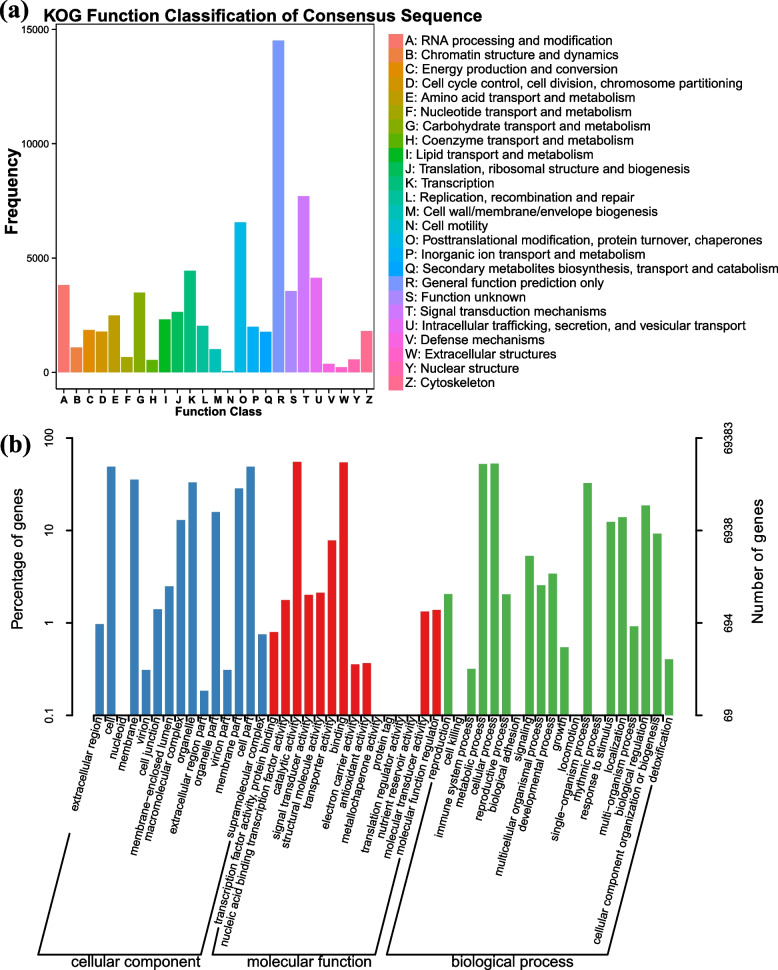
**a** KOG database annotation result distribution; **b** GO database annotation distribution histogram

A total of 311,258 CCS sequences in *G. sinensis* were read, with an average sequence length of 3408 and an average sequencing depth of 30X. Among them, the number of circular consensus sequencing (CCSs) was 256,015, accounting for 82.25%. After isoform sequence clustering, 141,905 identical sequences and 137,850 HQ sequences (97.14%) were obtained. A total of 93,668 open reading frames (ORFs) were obtained (Table S1). BUSCO estimated 1113 completeness. In total, 92,358 genes were annotated using the NR database, SwissProt annotated 68,784, and KEGG annotated 42,205. A total of 221 genes were annotated to the ko00900 (terpenoid backbone biosynthesis) pathway, and Hmmsearch annotated a total of 315 cytochrome P450 monooxygenases (*CYP450s*) and 147 uridine diphosphate-glycosyltransferases (*UGTs*). The KOG database annotated 63,739 (Fig. 1a). The KOG analysis revealed that 14,517 genes were annotated in the general function prediction only, and 7,706 genes in the signal transduction mechanisms. The GO database annotated 68,784 (Fig. 1b). The results of the GO enrichment analysis showed that the genes were primarily enriched in cellular processes (36,606, biological process), cell (34,404, cellular component), and catalytic activity (38,373, molecular function). Based on the alignment of sequence homology, 16,024 (74.33%) sequences were found against *Cajanus cajan*; 14,739 (15.97%) sequences were found against *Glycine max*, followed by *Lupinus angustifolius* (9754, 10.57%), *Glycine soja* (4262, 4.62%), and *Cicer arietinum* (4032, 4.37%). A total of 29,255 (31.70%) sequences were homologous to those of other species.

### Transcription factor and LncRNA identification

**Fig. 2:**
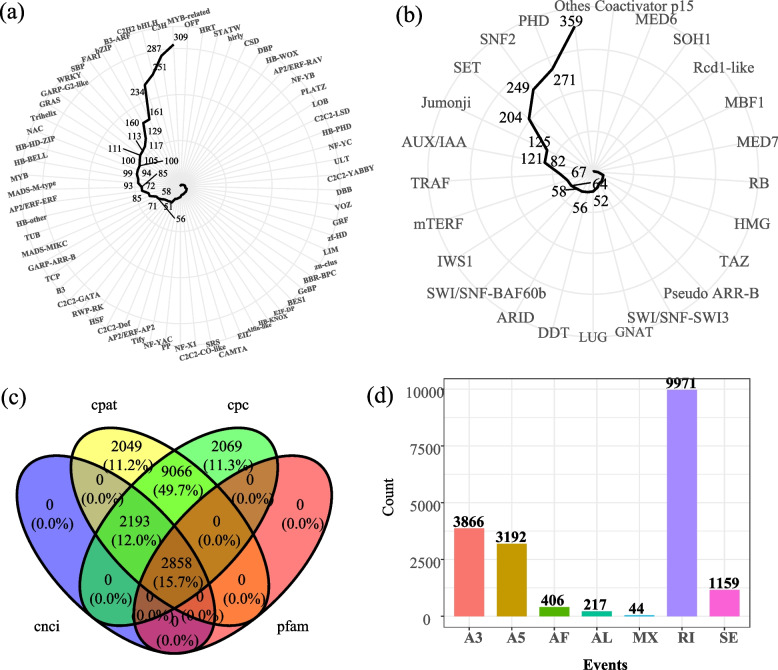
**a** Prediction of TFs of *G. sinensis* transcripts; **b** Prediction of TRs of *G. sinensis *transcripts; **c** Venn diagram of the number of predicted LncRNAs; **d** types and numbers of different AS events detected in *G. sinensis*

By predicting non-redundant transcripts, a total of 3645 transcripts were predicted to be TFs, and 1945 transcripts were predicted to be transcript regulators (TRs). A total of 309 transcripts were predicted to be MYB-related TFs, 287 transcripts were predicted to be C3H TFs, followed by bHLH (251) and C2H2 (234) (Fig. 2a). A total of 271 transcripts were predicted to be PHD TRs, and 249 transcripts were predicted to be SNF2 TRs. A total of 204 transcripts were predicted to be SET TRs (Fig. 2b). 2858 (15.7%) transcripts were simultaneously screened as lncRNAs (Fig. 2c) by CNCI, CPC, Pfam-scan, and PLEK. In total 81,182 SSR loci were identified, mono nucleotide motifs (52,741, 64.97%) were the most abundant type of SSR locus. As *G. sinensis* had no reference genome, Congent was used to divide the full-length transcripts into clustering families and reconstruct each family into one transcript model or several full-length unique transcript models (UniTransModels) based on K-mer clustering and De Bruijin graph methods. As a result, a total of 59,067 UniTranModels were yielded. A total of 18,855 AS events were identified (Fig. 2d), including seven AS types (Alternative 5'/3' splice sites (A5/A3), Alternative First/Last Exons (AF/AL), Mutually exclusive exons (MX), Retained intron (RI) and Skipped exon (SE)). Retained introns (RIs) (9971, 52.88%) were the majority of AS events.

### Gene selection pressure analysis

The numbers of one-to-one orthologous genes-matching *G. sinensis* against *G. delavayi*, *G. japonica*, *G. velutina*, *G. australis*, *G. microphylla*, *Gymnocladus chinensis*, and *Senna tora*—were 8060, 8916, 8956, 8926, 8978, 9281 and 4328, respectively. The mean Ka, Ks, and Ka/Ks ratios of the *G. sinensis* and *Senna tora* pairing were 0.129, 0.547, and 0.241; for the *G. sinensis* and *Gymnocladus chinensis* pairing the mean values were 0.063, 0.211, and 0.178, respectively.

**Fig. 3:**
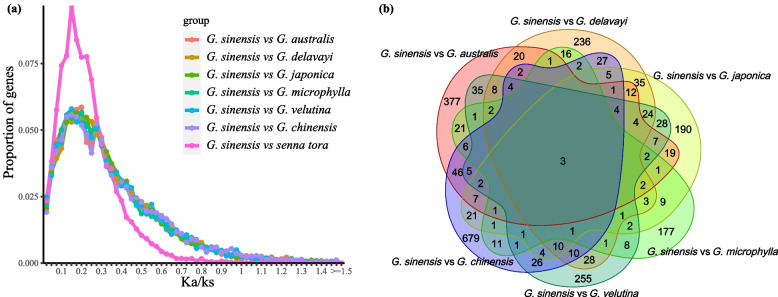
Comparative analysis of the one-to-one orthologous genes between *G. sinensis* and other species (**a**) Distribution of Ka/Ks ratio between groups; **b** Venn diagram of orthologous genes with Ka/Ks > 1

After calculating and filtering the Ka/Ks results, 264 pairs with Ka/Ks > 1 were identified between *G. sinensis* and *G. australis*, 294 pairs between *G. sinensis* and *Gymnocladus chinensis* were identified (Fig. 3a). The commonality analysis of *G. sinensis* and five other species with Ka/Ks > 1 revealed that transcript 66443 (ADP-ribosylation factor, *ARF*) and transcript 35059 (NADH-ubiquinone oxidoreductase chain 2, *ND2*) were in all pairwise combinations (Fig. 3b). A total of five orthologous genes with Ka/Ks > 1 were observed between *G. sinensis* and *Senna tora*; these were transcript 70110 (hypothetical protein VITISV_015170), transcript 75959 (autophagy-related protein 3-like isoform X2, *ATG3*), transcript 95937 (phosphoenolpyruvate carboxylase kinase 1-like, *PPCK1*), transcript 110936(xyloglucan endotransglucosylase/hydrolase protein 2, *XTH2*), and transcript 125127 (hypothetical protein TorRG33 × 02_055990).

### Analysis of codon usage bias in *Gleditsia* genus

**Fig. 4:**
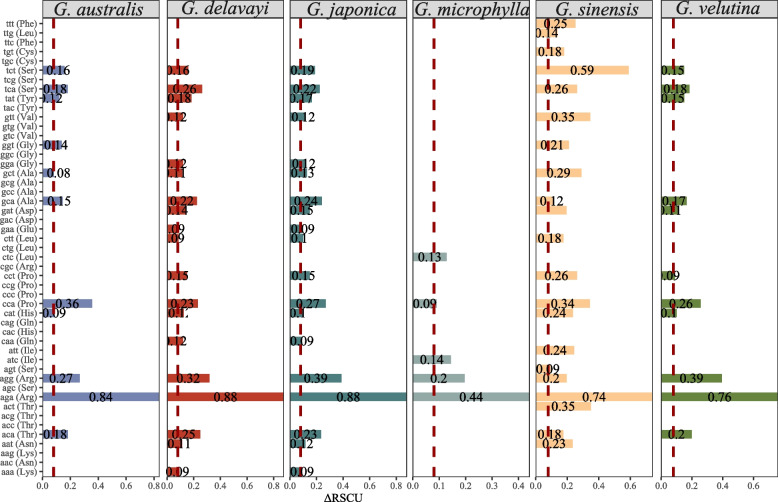
Putative optimal codons distribution of the transcriptome in Gleditsia. Note: ΔRSCU represents the difference between the high-expressed gene RSCU and the low-expressed gene RSCU; the red dashed line corresponds to the Y axis of 0.08

The CDS sequences from the *Gleditsia* genus transcriptomes were extracted, the numbers of CDS in *G. australis*, *G. delavayi*, *G. japonica*, *G. microphylla*, *G. sinensis*, *G. velutina*, *Gymnocladus chinensis*, were 77,181, 46,984, 64,712, 51,590, 69,945, 73,641, 65,417, respectively. The interval distribution frequency analysis of the CDS lengths found that as the sequence length increased, the frequency distribution gradually tended to be flat. Analysis of the GC content of different positions and average GC content showed that the GC content of the *Gleditsia* species was between 39.09% and 50.76%, which indicates that the code for the encoded protein by the *Gleditsia* genus prefers A/T base. PR2 bias plot analysis showed the 3rd position of the codons of the genus *Gleditsia* prefers T/G bases. The optimal number of codons for *G. australis*, *G. delavayi*, *G. japonica*, *G. microphylla*, *G. sinensis*, *G. velutina* was 12, 20, 20, 6, 21, 11, respectively. Among them, AGA (Arg), AGG (Arg), and CCA (Pro) appeared as universal optimal codons in most *Gleditsia* species (Fig. 4).

### Selection of reference genes for RT-qPCR

Eight candidate reference genes were amplified by PCR in cDNA samples which extracted from different tissues. Single bright bands were amplified from all cDNA samples. All genes showed single peaks in RT-qPCR melting curves in different samples (Figure S1), indicating that the cDNA amplification product was single. *EIF5A* was selected as the reference gene based on its expression stability measured by Cq.

### Identification of metabolic pathway related genes in triterpenoid saponins

**Fig. 5:**
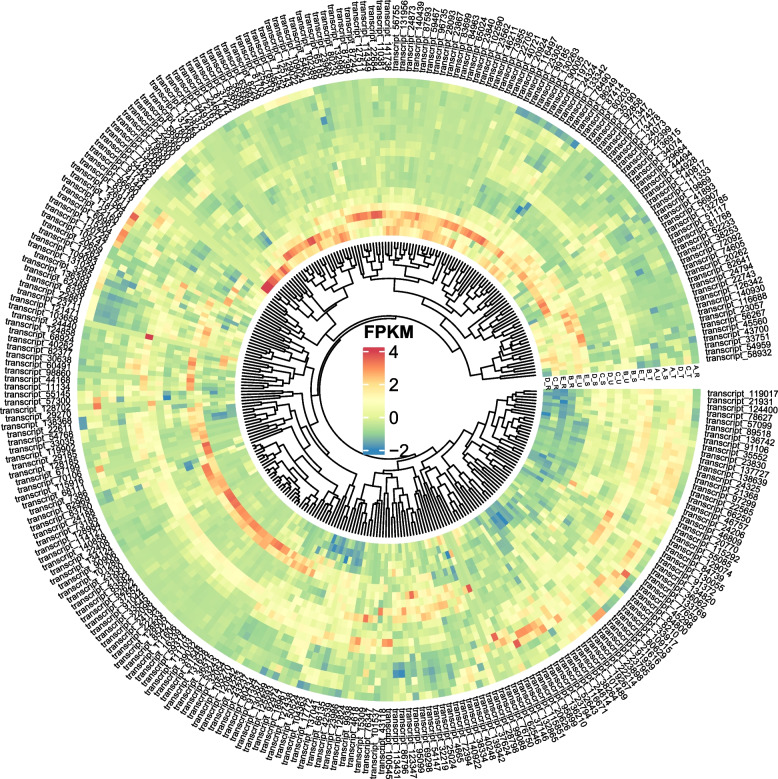
the heatmap of FPKM expression of *CYP450s*. Note: The expression data comes from quantitative data of the transcriptomes of different stages of *G. sinensis *thorn development (Project accession: PRJNA869136. Four different parts of *G. sinensis* at five developmental stages (2 DAG (labeled term A), 3 DAG (labeled term B), 7 DAG (labeled term C), 8 DAG (labeled term D), and 14 DAG (labeled term E)) were subjected to transcriptome sequencing (RNA-seq). The four different parts were the thorn stem segments (labeled S), the non-thorn stem segments (labeled U), the top of the stem (labeled T), and the tip of the root (labeled R), respectively

We obtained 315 *CYP450s *(Figure S2) and 147 *UGTs* (Figure S3) from the full-length transcriptome through against the pfam daftabase. Through alignment with the existing transcriptome (*acc*: PRJNA869136), and comparative quantification of sample expression, a heatmap of *CYP450-related* genes was generated (Fig. 5), revealing that *ent-kaurenoic acid oxidase 2* (a member of *CYP450*, transcript_103658) demonstrates significantly elevated expression during the thorn primordium stage (B_S), suggesting its potential involvement in thorn development. The expression of *UGTs* exhibited tissue specificity (Figure S4), with certain genes showing relatively high expression in the root, stem, apical region, and thorn. This observation suggests that the genes involved in the biosynthesis of triterpenoid saponins may vary across different parts of the *G. sinensis* plant.

### Cloning and subcellular localization of *GsIAA14*

**Fig. 6:**
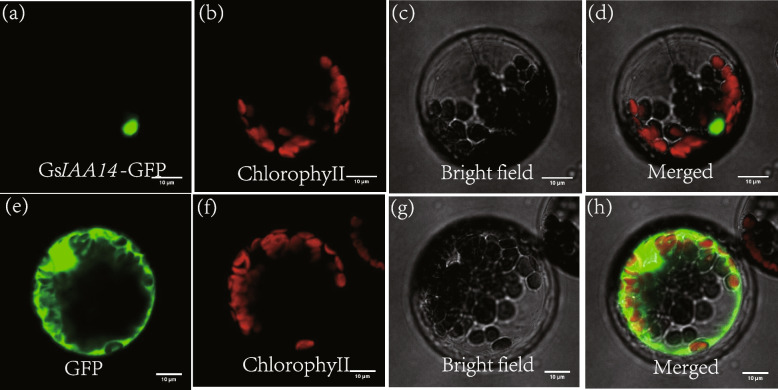
The subcellular localization of GsIAA14 by the GFP-fusion protein in pBWA(V)HS-GLosgfp. Note: **a**-**d** Left to right were the target protein fluorescence channel, chloroplast channel, bright field, superimposed map; **e**–**h**: the empty vector control were the fluorescence channel, the chloroplast channel, the bright field, and the superimposed map, respectively

For the identification of the *IAA/AUX* gene family, a total of 112* IAA/AUX *genes were identified in *G. sinensis*, and *AUX28* (transcript_136770) was highly expressed in all samples (Figure S5). *IAA14* (transcript_53903) and *IAA28* (transcript_136770) showed relatively conserved motifs. We randomly selected and cloned the full-length of *IAA14* using specific primers and sanger sequencing revealed its sequence was consistent with third-generation sequencing. The vector pBWA(V)HS-*GsIAA14*-GFP fused with GFP at the C-terminus was transformed into *Arabidopsis* protoplasts (Fig. 6). The green fluorescence of the fused GFP of 35S::*GsIAA14* was observed in the nucleus, and the fluorescence was bright, suggesting that *GsIAA14* was located in the nucleus.

## Discussion

The lack of a *G. sinensis* reference genome hinders the research of molecular biotechnology in genotype selection and molecular breeding. Pacbio SMRT sequencing makes it possible to obtain the full length of the transcriptome. In this study, to determine the number of large transcripts, the samples used to prepare RNA covered the different organs and physiological states of male and female *G. sinensis* plants. We obtained the original Binary Alignment Map (BAM) file of approximately 67 Gb. A total of 311,258 CCS sequences in *G. sinensis* were obtained, with an average sequence length of 3408, 256,015 full-length reads were obtained. Clustering the FLNC sequences yielded 141,905 consensus sequences and refining the consensus sequences produced a total of 137,850 HQ consensus sequences; removing redundancies reduced these to 95,183 transcript sequences. Of these, 3645 transcripts were predicted to be transcription factors, 1945 transcripts were predicted to be transcript regulators, and 2858 transcripts were simultaneously screened as lncRNAs. A total of 18,855 AS events were identified, RIs (9971, 52.88%) were the majority of AS events.

The word “saponin” is derived from the Latin word “sapo,” literally meaning soap, and the pod of *G. sinensis* has been used as "soap" for thousands of years [16, 17]. Triterpenoid saponins are one of the most important components of *G. sinensis* [18]. Triterpene saponin biosynthesis is mainly divided into three parts: precursor formation, skeleton construction, and later modification [19]. Saponin biosynthesis begins with the generation of triterpenoid/sterol aglycone via the mevalonic acid (MVA) pathway or methyl erythritol pathway (MEP; [17]. Candidate Cytochrome P450 monooxygenases (*CYP450*) further modify the triterpene backbone to produce triterpenes of diverse structure, and some triterpenes introduce glycosyl groups under the action of uridine diphosphate (UDP)-glycosyltransferases (*UGTs*), and produce complex and diverse triterpene saponins [20, 21]. Many of the tissue-specific genes expressed in *G. sinensis* pods are involved in the biosynthetic pathway of flavonoids, most of which have UGT activity [22]. The *UGT* and *CYP450* gene families of *Arabidopsis* contain 107 and 252 members, respectively [23, 24]. In *Psammosilene tunicoides*, a total of 114 putative *CYP450s* were recognized through PacBio SMRT transcriptome data, *PtCYP72A219* showed the largest increase compared to controls after 8 h of SA applications [25]. 6 *CYPP450s *and *24UGTs* related to the biosynthesis of triterpenoid saponins were discovered with high transcriptome expression through the weighted gene co-expression network analysis [26]. We obtained 315 *CYP450s* and *147 UGTs* from the full-length transcriptome through against the pfam daftabase. Compared with the data from the de novo RNA-seq transcriptome in previous studies (*CYP450* and *UGTs*, encoded by 37 and 77 unigenes, respectively [22]. From an another research, 136 *CYP450s* and 77 *UGTs* were annotated, seven P450s and one *UGT* that were highly expressed in the fruit of *G. sinensis* were identified as candidate genes involved in the biosynthesis of triterpenoid saponins [22]. This implies that PacBio SMRT sequencing data in this study showed strong gene coverage. Through alignment with the existing transcriptome (*acc*: PRJNA869136), *ent-kaurenoic acid oxidase 2* (a member of *CYP450*, transcript_103658) demonstrates significantly elevated expression during the thorn primordium stage, suggesting its potential involvement in thorn development. The expression of *UGTs* exhibited tissue specificity (Figure S), with certain genes showing relatively high expression in the root, stem, apical region, and thorn. This observation suggests that the genes involved in the biosynthesis of triterpenoid saponins may vary across different parts of the *G. sinensis* plant.

For non-model species without genome sequencing, the use of comparative transcriptome alignment to obtain orthologous genes and gene selection pressure analysis is a quick way to identify evolutionary patterns between species. The transcriptome of *Pinus kesiya* var. *langbianensis* was sequenced using RNA-seq and assembled by Trinity, and all pairwise orthologs were identified by comparative transcriptome analysis [27]. 54 unigenes were subjected to positive selection (Ka/Ks > 1) between *Agave tequilana* and *Agave sisalana* [28]. In this study, we identified genes homologous with the *G. sinensis* transcriptome obtained by PacBio SMRT and other assembled transcriptome data by RNA-seq. Ka/Ks > 1 indicates that these genes have been involved in positive selection during evolution. *ATG3* (transcript_75959), *PPCK1* (transcript_95937), *XTH2* (transcript_110936), and two other unknown functional genes were observed in common between *G. sinensis* and *Senna tora* with Ka/Ks > 1. The commonality analysis of *G. sinensis* and the other varieties with Ka/Ks > 1 found that the *ARF *and *ND2* were in all pairwise combinations. ARFs belong to one group within the Ras superfamily of GTP-binding proteins [29]. Three *ARFs* (*PvArf1*, *PvArf-B1C*, and *PvArf-related*) contribute to salinity tolerance in transgenic *Panicum virgatum* [30]. *TaARFs* are induced in response to abiotic and biotic stresses in *Triticum aestivum* [31]. The NADH:ubiquinone oxidoreductase (complex I) is the first enzyme in the respiratory chain and the entry point for most electrons [32]. Complex I of the respiratory chain has several remarkable features in plants; in particular, many of its subunits are encoded by the mitochondrial genome and it is indirectly involved in photosynthesis [33]. This indicates that the stress-related genes had been positively selected during the evolution of different *Gleditsia* species. CUB refers to differences in the relative frequency of synonymous codons for individual amino acids in protein coding sequences [34]. The research on the preference of CUB plays an important role in the phylogenetic development relationship and the application of foreign gene improvement to transgenes. Codon usage of highly expressed genes was selected in evolution to maintain the efficiency of global protein translation [35]. The third position of the codon of genus *Gleditsia* prefers the T/G base. In 13 high-frequency codons among different citrus, 11 of them were the same [36]. Through the optimal codon screening, the optimal codon numbers for *G. australis*, *G. delavayi*, *G. japonica*, *G. microphylla*, *G. sinensis*, *G. velutina* was 12, 20, 20, 6, 21, 11, respectively. Among them, AGA, AGG, and CCA were selected as universal optimal codons in *Gleditsia* species. Meanwhile, screening of stably expressed internal reference genes can lay the foundation for accurate gene expression. In order to detect applicable reference genes, based on the coefficient of variation in expression by searching for literature and identifying homologous genes, eight candidate genes (*Actin*, *Hsp90*, *Hsp70*, *RPL9*, *18S*, *28S*, *EIF5A*, *EF1*) were selected, *EIF5A* as the reference gene based on its expression stability measured by Cq.

In order to verify the reliability of PacBio SMRT transcriptome assembly sequence, we randomly selected *AUX/IAA *gene family for identification, a total of 112 *IAA/AUX *genes were identified in *G. sinensis*, and *AUX28* (transcript_136770) was highly expressed in all samples (Figure S1). *IAA14* (transcript_53903) and *IAA28* (transcript_136770) showed relatively conserved motifs. *IAA28* and *IAA18* were identified as mobile transcripts in the cortex of the model plant *Arabidopsis*, and micrografting experiments confirmed that the IAA transcripts produced in the vascular tissue of mature leaves were subsequently transported to the root system [37]. *MpIAA14* of apple was detected to be able to detect long-distance transportation through the grafting junction [38]. We randomly selected and cloned the full-length of *IAA14* using specific primers and sanger sequencing revealed its sequence was consistent with PacBio SMRT sequencing. Subcellular localization showed that *GsIAA14* was located in the nucleus (Fig. 6). This indicates that using PacBio transcriptome data can quickly clone and obtain the full length of the target gene for later functional verification analysis.

## Conclusions

In the present study, we performed the PacBio SMRT sequencing of *G. sinensis* with high coverage. A total of 95,183 non-redundant transcript sequences were obtained, of which 93,668 contained complete open reading frames and 2,858 were long non-coding RNAs. 315 CYP450s and 147 UGTs were recognized through the PacBio SMRT transcriptome. Orthologous genes were identified between different species. Stress-related genes had been positively selected during the evolution of different *Gleditsia* species. AGA, AGG, and CCA were selected as universal optimal codons in *Gleditsia* species. *EIF5A* was selected as a suitable fluorescent quantitative reference gene. Randomized selection of *GsIAA14* for full-length cloning verified the reliability of the PacBio transcriptome assembly sequence.

## Materials and methods

### Experimental materials

To investigate the genetic information of *G. sinensis* as broadly as possible, full-length transcriptome test samples were collected from different parts of the male and female plants. The female plant material was selected from a 20-year-old female *G. sinensis*, Lushan Town (25°56ʹ56.7ʺ N, 106°30ʹ18.4ʺ E), Huishui County, Guizhou Province, China. The samples included branch segments, stem cambium, secondary lateral roots, leaf buds, new leaves, mature leaves, inflorescence primordia, small flower spikes (inflorescence formation period), unopened female flowers (single flower organs), developing and forming single-flower organs, pods, thorn buds, tender thorns, and seeds. Male plant material was selected from the Tianhetan Plantation Park (26°38ʹ29.8ʺ N, 106°13ʹ57.1ʺ E) in Guiyang City, Guizhou Province, China. These samples included branch segments, stem cambium, secondary roots, leaf buds, new leaves, mature leaves, flower primordia, small flower spikes (inflorescence formation period), single flower organ development and formation, thorn buds, and tender thorns. All samples were wrapped in aluminum foil, quickly frozen in liquid nitrogen, and then transferred to a refrigerator at − 80°C for storage. These samples were used for PacBio SMRT sequencing.

To identify genes that are subject to positive selection pressure, we collected other various species of *Gleditsia* in China, including *G. australis* (Conghua district, GuangDong Province), *G. japonica* (Zhijing city, Guizhou Province), *G. microphylla* (Zhijin city, Guizhou Province)), *G. japonica* var. *delavayi* (Xinyi city, Guizhou Province), and *G. japonica* var. *velutina* (Changsha, Hunan Province). In addition, *Gymnocladus chinensis* Baill (*Fam.*: *Leguminosae*; *Gen.*: *Gymnocladus*) (Duyun city, Guizhou Province) was collected as the outer group. The collected seeds were subjected to germination treatment, and functional new leaves of different species after one month of cultivation were used for RNA extraction. High-throughput mRNA sequencing of these different *Gleditsia* species and *Gymnocladus chinensis* was performed using RNA-seq.

### RNA extraction and library preparation

RNA integrity was assessed by agarose gel electrophoresis, while its integrity number (RIN) was measured using an Agilent 2100 (Agilent Technologies, Santa Clara, California, USA). The RNA extraction quality and concentration of all samples was satisfactory (A260/280 = 2.0–2.2; A260/230 = 1.8–2.2; 28S/18S = 1.4–2.7; Rin ≥ 8.0). The mRNA was enriched with Oligo (dT) magnetic beads.

For the PacBio SMRT sequencing, total RNA from different tissues was pooled in equal amounts, and 2 µg of the pooled RNA was used for cDNA synthesis and SMRT library construction. the library construction process was as follows: A SMARTer PCR cDNA Synthesis Kit (Takara) was used to synthesize full-length cDNA from the mRNA. Size selection was carried out, and 1–6 kb fractions were collected. To obtain a sequencing library, PCR amplification of full-length cDNA, end-repair of full-length cDNA, connection of the SMRT dumbbell linker, and exonuclease digestion were performed. After the library was qualified, it was sequenced using the PacBio platform (Pacific Biosciences, Menlo Park, CA, USA). The PacBio raw bam file was deposited in the National Center for Biotechnology Information (NCBI) Sequence Read Archive (SRA) (SRA; BioProject Accession PRJNA722800).

For the Illumina sequencing, the mRNA was added to fragmentation buffer and cut into short fragments. Using mRNA as a template, cDNA was reverse-transcribed using six-base random primers. The double-stranded cDNA samples were purified, end-repaired, added with poly(A) tails, and then ligated to sequencing adapters to create cDNA libraries. After the libraries passed the quality test, qualified libraries were sequenced using an Illumina HiSeq machine with paired-end 150 bp reads. The raw reads generated from Illumina sequencing were deposited in the NCBI SRA database (*acc.* PRJNA722818).

### Statistics, quality control, and annotation of raw sequencing data

The raw reads from the PacBio platform were filtered using SMRTLink (https://www.pacb.com/support/software-downloads/). SMRT CCS with default parameters to obtain post-filter polymerase reads. After CCS quality control, classification, and clustering, low-quality and high-quality isoform sequences were obtained with an accuracy of greater than 99%. The analysis process for obtaining the full-length transcriptome [39]: For full-length sequence identification, all original sequences were converted to CCS sequences according to the adaptor, then divided into full-length and partial sequences based on the location of the 3ʹ primer, 5ʹ primer, and poly(A); For isoform-level sequencing, the IsoSeq module in the SMRTLink software was used to group similar sequences within the full-length non-chimeric sequence (*i.e.*, multiple copies of the same transcript) into a cluster, with each cluster having a consensus isoform. The consistent sequences in each cluster were further corrected, and high-quality (HQ; accuracy greater than 99%) and low-quality transcripts were obtained. CD-HIT v4.8.1 [40] was used to remove redundant sequences from HQ transcripts. Benchmarking Universal Single-Copy Orthologs (BUSCO; [41] estimated the transcript integrity of some conserved genes in related species. According to the priority order of NCBI non-redundant (NR) protein sequences, Swiss-Prot, Kyoto Encyclopedia of Genes and Genomes (KEGG) database [42], and EuKaryotic Orthologous Groups (KOG) databases/tools, the transcripts were subjected to BLAST queries (*e-value* < 0.00001). To identify the gene family, using the Hmmer v3.3.1 software [43] to against Pfam.

### Detection of alternative splicing, simple sequence sepeats and LncRNAs

To obtain alternative splicing (AS) events for *G. sinensis*, Coding GENome reconstruction tool (Cogent v3.9, https://github.com/Magdoll/Cogent) was used to reconstruct the coding genome [14]. Error-corrected non-redundant transcripts (transcripts before Cogent reconstruction) were mapped to UniTransModels using minimap2 [44]. AS events were detected with SUPPA (https://github.com/comprna/SUPPA) using default settings. The software MISA was employed to identify SSRs with default settings. Putative protein-coding RNAs were filtered using minimum-length and exon-number thresholds. ESTScan software [45] was used to predict the coding region (sequence direction 5ʹ to 3ʹ) if none of the above protein databases produced a match. Analysis was performed using the Coding Potential Assessment Tool (CPAT), Coding-Non-Coding Index (CNCI), Coding Potential Calculator (CPC), and protein-family structure domain analysis (Pfam) [46–49].

### Identification of orthologous gene groups and calculation of Ka/Ks ratios

The transcriptomes of the five different *Gleditsia* species were quality-controlled using the fastp v 0.22.0 software [50]. Clean reads were assembled using Trinity v2.15.1 software [51] and the assembled sequences combined and clustered using CD-HIT v4.8.1 [40]. OrthoFinder software [52] with default parameters was used to identify orthologous genes in the full-length transcriptomes of *G. sinensis* and other transcriptomes of *Gleditsia* and *Senna tora*. The *Senna tora* genome was downloaded from the NCBI database (https://www.ncbi.nlm.nih.gov/genome/?term=Senna+tora) [53]. Sequences of each one-to-one orthologous gene were aligned using ParaAT [54]. The non-synonymous substitution rates (Ka), synonymous substitution rates (Ks), and Ka/Ks ratios for each orthologous pair were calculated using KaKs Calculator 2.0 [55] with the YN algorithm. Genes were classified according to previous studies [56, 57]: genes with Ka/Ks < 0.5, were treated as under purifying selection, 1 > Ka/Ks > 0.5 indicated genes under weak positive selection, and Ka/Ks > 1 indicated genes under strong positive selection (that had previously experienced positive selection).

### Analysis of codon usage bias

Extracted the full-length coding sequences exceeding 300 bp, with an ATG start codon, a stop codon (TGA/TAG/ TAA). The nucleotide compositions at the third position (A3s, U3s, C3s and G3s), GC content at third codon positions (GC3s), codon adaptation index (CAI), Codon Bias Index (CBI), effective number of codon (ENC) were determined with CodonW v1.4.4 software [58]. The length, GC content of the CDSs, and relative synonymous codon usage (RSCU) were calculated by the seqinr [59]. We selected 10% of the total genes with extremely high and low CAI values which were regarded as the high and low expression gene datasets, respectively. Optimal codons were defined as those positive RSCU ≥ 1.0 and ∆RSCU (RSCU_mean highly expressed CDSs_—RSCU_mean lowly expressed CDSs_) ≥ 0.08 [60, 61]. Besides, six species of *Gleditsia* have the same optimal codon, which was selected as the universal optimal codon in the genus of *Gleditsia*.

### Selection of reference genes for RT-qPCR

To investigate the expression of candidate reference genes at different developmental stages of *G. sinensis*, homologous genes were identified by searching the literature, and 8 candidate genes (*Actin*, *Hsp90*, *Hsp70*, *RPL9*, *18S*, *28S*, *EIF5A*, *EF1alpha*) were selected based on the coefficient of variation of their expression levels (The reference gene primers are shown in Table S2). RNA were extracted from the branches, stem cambium, secondary roots, leaf buds, new leaves, mature leaves, inflorescence primordia, young flower clusters (during inflorescence formation) and unopened female flowers (single flower organs), formation of single flower organs, early stage of fruit formation, thorn buds, and young thorns of female trees and male trees. Total RNA (1 µg) was used for cDNA synthesis using the Prime Script™ RT reagent Kit (Takara Biotechnology, China). Prior to RT-qPCR validation, the specificity of the primers was verified by 1% agarose gel electrophoresis. The amplification reaction was performed with 2 µl of cDNA template, 0.5 µl of upstream and downstream primers, 10 µl of qPCR mix buffer, and 7 µl of ddH_2_O, for a total volume of 20 µl. RT-qPCR was performed using the LightCycler® 480 Instrument II (Roche). The PCR program consisted of a denaturation step at 95°C for 3 min, followed by 40 cycles of denaturation at 94°C for 10 s, annealing at 59°C for 10 s, and extension at 72°C for 40 s. The expression stability of the eight genes was ranked using NormFinder software [62].

### Identification of metabolic pathway related genes in triterpenoid saponins

Candidate Cytochrome P450 monooxygenases (*CYP450*) and uridine diphosphate (UDP)-glycosyltransferases (*UGTs*) were predicted through the pfam database annotation. Mafft [63] was used to align the sequences. Fastree software [64] was used to construct sequence evolution trees. MEME suite (https://meme-suite.org/meme/) [65] was used to identify gene motif features online, with a maximum of 10 motifs, a minimum motif length of 6, a maximum motif length of 50, and a minimum of 2. We download the transcriptomes of different stages of *G. sinensis* thorn development (Project accession: PRJNA869136). The fastp [50] software was used as quality control raw data, Bowtie2 (https://bowtie-bio.sourceforge.net/bowtie2/index.shtml) and RSEM (https://deweylab.github.io/RSEM/) were used for comparison and expression quantification, and the expression value was calculated using FPKM (Fragments Per Kilobase of transcript per Million mapped reads).

### Reliability of PacBio SMRT assembled sequence

In order to verify the reliability of the third-generation transcriptome assembly sequence, we selected the IAA/AUX gene family for sequence motif identification and randomly selected *GsIAA14* for cloning based on the motif and expression quantity. Using the *G. sinensis* PacBio SMRT assembled data in this study (accession No.: PRJNA722800) and the Aux/IAA HMM (Hidden Markov Model) file (PF02309, http://pfam-legacy.xfam.org/), used the HMM file as a seed to perform HMM searching (*e-value* < 1e-20) in the protein database encoded by *G. sinensis* CDS. Non-redundant protein IDs were extracted, and corresponding gene ID sequences and length information were collected. We downloaded the SRA data from NCBI SRA database (Project accession: PRJNA946805). Different tissues of male and female *G. sinensis*, including flowers, leaves, leaf primordia, thorns, and main roots were collected as mixed samples for RNA extraction. Total RNA was extracted from the mixed tissue samples using the Trizol reagent kit, RNA integrity was checked by agarose gel electrophoresis. Cloning was carried out with the PCR (Cloning primers: *GSsIAA14*( +):ATGGCAACTTTGCTGGGGAAGGAGG;(-):TCAGCTTCTGCTTTTGCATTTTTCC). Golden gate technology was used to construct overexpression vectors. A 50 µl PCR reaction was performed to amplify the target fragment using the overexpression vector primers (pBWA(V)HS-GsIAA14( +):cagtGGTCTCacaacatggcaactttgctggggaa;pBWA(V)HS-GsIAA14(-)cagtGGTCTCatacagcttctgcttttgcattttt), and the gel-extracted product was sequenced and confirmed before being ligated to the vector pBWA(V)HS-ccdb-GLosgfp. Subcellular localization was carried out using *Arabidopsis* protoplasts, and observation was performed using confocal laser scanning microscopy (Nikon C2-ER, Nikon).

### Supplementary Information

**Additional file 1.**

## Data Availability

The PacBio raw bam file in this study have been deposited in the NCBI SRA database (accession BioProject: PRJNA722800). The raw reads generated from Illumina sequencing have been deposited in the NCBI SRA database (accession BioProject: PRJNA722818).

## Abbreviations

CCS: Circular consensus sequencing
CUB: Codon usage bias
CYP450s: Cytochrome P450 monooxygenases
*Gleditsia sinensis*: *G. sinensis*
HMM: Hidden Markov Model
Ka: Non-synonymous substitution
Ks: Synonymous substitution
NCBI: National Center for Biotechnology Information
ORF: Open reading frames
RSCU: Relative synonymous codon usage
RNA-Seq: Second-generation sequencing technology
RT-qPCR: Real-time quantitative PCR
SRA: Sequence Read Archive
TF: Transcription factor
TR: Transcript regulator
SMRT: Single-molecule real-time
UGTs: Uridine diphosphate-glycosyltransferases
