# Supplementary material for: Full-length transcriptome characterization and comparative analysis of Gleditsia sinensis
Source: BMC Genomics. 2023 Dec 8;24:757. doi: 10.1186/s12864-023-09843-y (PMC10709882; doi:10.1186/s12864-023-09843-y)
Supplement: Supplementary file 1 — Additional file 1. [file 12864_2023_9843_MOESM1_ESM.zip › Supplementary/FigureS3.pdf]

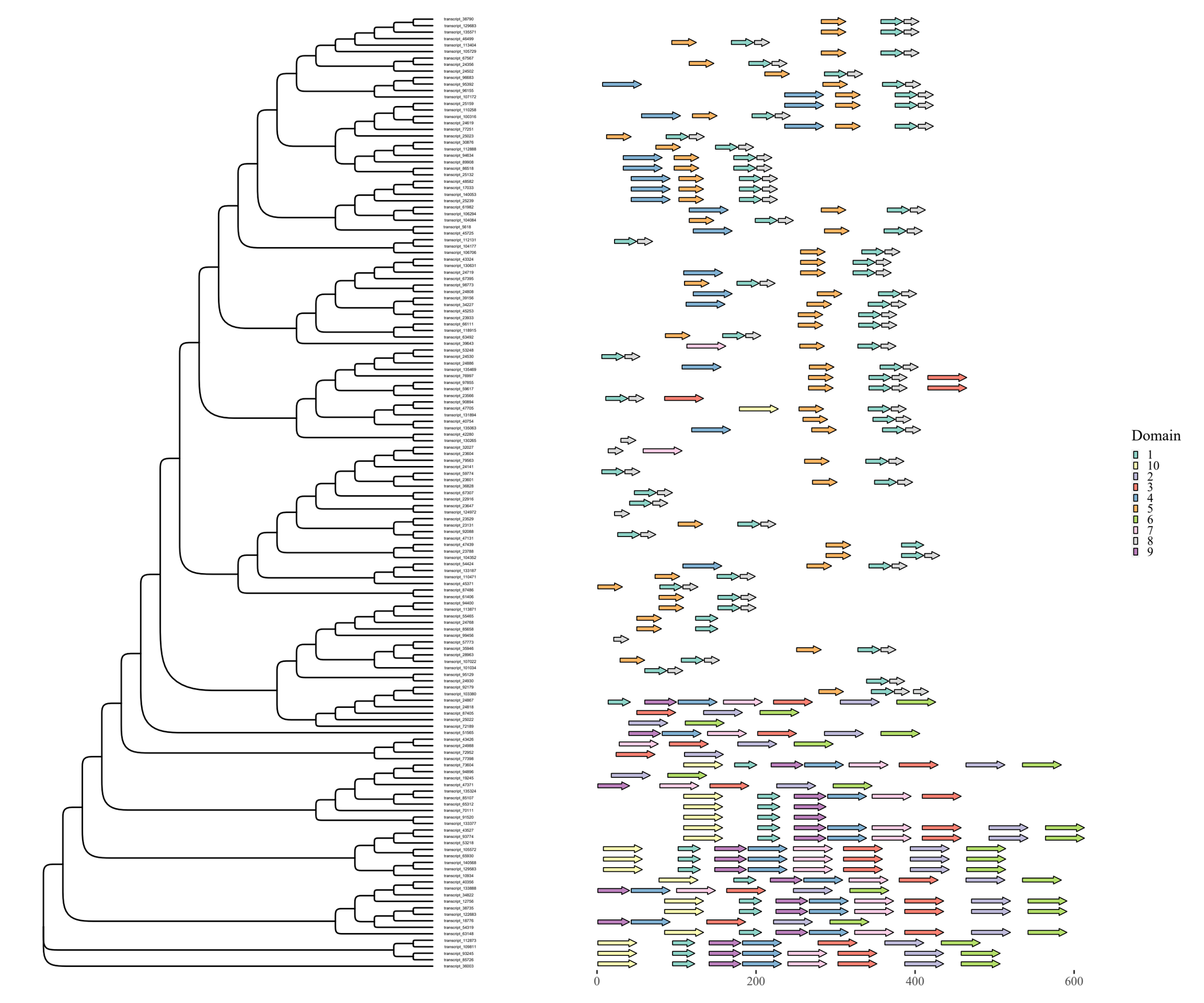
transcript_38790
transcript_129683
transcript_135571
transcript_46499
transcript_113404
transcript_105729
transcript_67567
transcript_24356
transcript_24502
transcript_96683
transcript_95392
transcript_96155
transcript_107172
transcript_25159
transcript_110258
transcript_100316
transcript_24619
transcript_77251
transcript_25023
transcript_30876
transcript_112888
transcript_94634
transcript_89908
transcript_86518
transcript_25132
transcript_48582
transcript_17033
transcript_140053
transcript_25239
transcript_61982
transcript_106294
transcript_104084
transcript_5618
transcript_45725
transcript_112131
transcript_104177
transcript_106706
transcript_43324
transcript_130631
transcript_24719
transcript_67395
transcript_98773
transcript_24808
transcript_39156
transcript_34227
transcript_45253
transcript_23933
transcript_66111
transcript_118915
transcript_63492
transcript_39643
transcript_53248
transcript_24530
transcript_24886
transcript_135469
transcript_76997
transcript_97855
transcript_59617
transcript_23566
transcript_90894
transcript_47705
transcript_131894
transcript_40754
transcript_135063
transcript_42280
transcript_130265
transcript_32027
transcript_23604
transcript_79563
transcript_24141
transcript_59774
transcript_23601
transcript_36828
transcript_67307
transcript_22916
transcript_23647
transcript_124972
transcript_23529
transcript_23131
transcript_92088
transcript_47131
transcript_47439
transcript_23788
transcript_104352
transcript_54424
transcript_133187
transcript_110471
transcript_45371
transcript_87486
transcript_61406
transcript_94400
transcript_113871
transcript_55465
transcript_24768
transcript_85658
transcript_99456
transcript_57773
transcript_35946
transcript_28963
transcript_107022
transcript_101034
transcript_95129
transcript_24930
transcript_92179
transcript_103380
transcript_24867
transcript_24818
transcript_87405
transcript_25022
transcript_72189
transcript_51565
transcript_43426
transcript_24988
transcript_72952
transcript_77398
transcript_73604
transcript_94896
transcript_19245
transcript_47371
transcript_135324
transcript_85107
transcript_65312
transcript_70111
transcript_91520
transcript_133377
transcript_43527
transcript_93774
transcript_53218
transcript_105572
transcript_65930
transcript_140568
transcript_129583
transcript_10934
transcript_40356
transcript_133888
transcript_34822
transcript_12756
transcript_38735
transcript_122683
transcript_18776
transcript_54319
transcript_63148
transcript_112873
transcript_109811
transcript_93245
transcript_85726
transcript_36003
0
200
400
600
Domain
1
10
2
3
4
5
6
7
8
9
