# Supplementary material for: Full-length transcriptome characterization and comparative analysis of Gleditsia sinensis
Source: BMC Genomics. 2023 Dec 8;24:757. doi: 10.1186/s12864-023-09843-y (PMC10709882; doi:10.1186/s12864-023-09843-y)
Supplement: Supplementary file 1 — Additional file 1. [file 12864_2023_9843_MOESM1_ESM.zip › Supplementary/Supplementary Information.docx]

**Supplementary Information**

**Figure S1.** Screening of internal reference primers Note: a-h were RT-qPCR melting curves of 8 different genes.

**Figure.S2** Phylogenetic tree, conserved motifs of *CYP450s Note:* The left was a series of evolutionary trees; the right was the conserved motif of MEME;

**Figure.S3** Phylogenetic tree, conserved motifs of UGTs *Note:* The left was a series of evolutionary trees; the right was the conserved motif of MEME;

**Figure.S4** the heatmap of FPKM expression of *UGTs* *Note:* The expression data comes from quantitative data of grafted related transcriptome (NCBI Project *acc.*:[PRJNA869136](https://www.ncbi.nlm.nih.gov/bioproject/PRJNA869136)). Four different parts of *G. sinensis* at five developmental stages (2 DAG (labeled term A), 3 DAG (labeled term B), 7 DAG (labeled term C), 8 DAG (labeled term D), and 14 DAG (labeled term E)) were subjected to transcriptome sequencing (RNA-seq). The four different parts were the thorn stem segments (labeled S), the non-thorn stem segments (labeled U), the top of the stem (labeled T), and the tip of the root (labeled R), respectively.

**Figure.S5** Phylogenetic tree, conserved domains and expression analysis of *AUX/IAA* gene family *Note:* The left was a series of evolutionary trees; the middle was the conserved domain of MEME; the right was the heatmap of FPKM expression at the top of different seedlings; *G.au*: *Gleditsia australis*; *G.de*: *Gleditsia delavayi*; *G.fe*: *Gleditsia fera*; *G.ja*: *Gleditsia japonica*; *G.mi*: *Gleditsia microphylla*; *G.si*: *Gleditsia sinensis*; "/" means grafting, with scion in front and rootstock behind. The expression data comes from quantitative data of grafted related transcriptome (NCBI Project *acc.*:PRJNA946805).

Table.S1 Related indicators of PacBio SMRT transcriptome raw analysis data

| **Samples** | **PacBio SMRT of G. sinensis** |
| --- | --- |
| CCS Number | 311258 |
| Read Bases of CCS | 1060909840 |
| Mean Read Length of CCS | 3408 |
| Mean Number of Passes | 30 |
| Number of undesired primer reads | 33007 |
| Number of filtered short reads | 0 |
| Number of full-length non-chimeric reads | 256015 |
| Full-length non-chimeric percentage (FLNC%) | 0.8225 |
| Average consensus isoforms read length | 141905 |
| Number of polished high-quality isoforms | 3463 |
| Number of polished low-quality isoforms | 137850 |
| Number of isoforms | 95183 |
| Total sequence length of isoforms | 339499398 |
| Average sequence length of isoforms | 3566.8 |
| Maximum sequence length of isoforms | 17238 |

Table.S2 Internal reference gene screening primer list

| **Primer name** | **Primer sequence(5'-3')** | **function** |
| --- | --- | --- |
| *18S rRNA*(+) | GAATGGAACGGTCTGTATCT | Fluorescence quantitative internal reference primer screening |
| *18S rRNA*(-) | TTCTACCTTGCTCCTTCTTC |  |
| *28S*(+) | TTCGTCTTCGGCTTCTTC |  |
| *28S*(-) | ACATCAGTCCAGTGAGTCT |  |
| *ACTIN*(+) | GTGGTGGCTCAACTATGT |  |
| *ACTIN*(-) | TCCTCCAATCCAGACACT |  |
| *EF1alpha*(+) | AACGGTGATGCTGGTATG |  |
| *EF1alpha*(-) | CTCCTTCTTCTCTACATTCTTG |  |
| *EIF5A*(+) | CATGTGAATCGTACTGACTATC |  |
| *EIF5A*(-) | GGTCATCCTTGGTGTTCC |  |
| *HSP90*(+) | GCTGATGCTGACAAGAATG |  |
| *HSP90*(-) | ATCCTGTGAATCCTGTTACC |  |
| *RPL9*(+) | CCAATAGCAACAAGTCCATT |  |
| *RPL9*(-) | CCATCCAATATCAACTCATCC |  |
| *HSP70*(+) | CCAAGCAAGGTGTTCAGA |  |
| *HSP70*(-) | GAGCAACTTCAGTAGAGGTA |  |
