# Supplementary figures and images for: Full-length transcriptome characterization and comparative analysis of Gleditsia sinensis
Source: BMC Genomics. 2023 Dec 8;24:757. doi: 10.1186/s12864-023-09843-y (PMC10709882; doi:10.1186/s12864-023-09843-y)

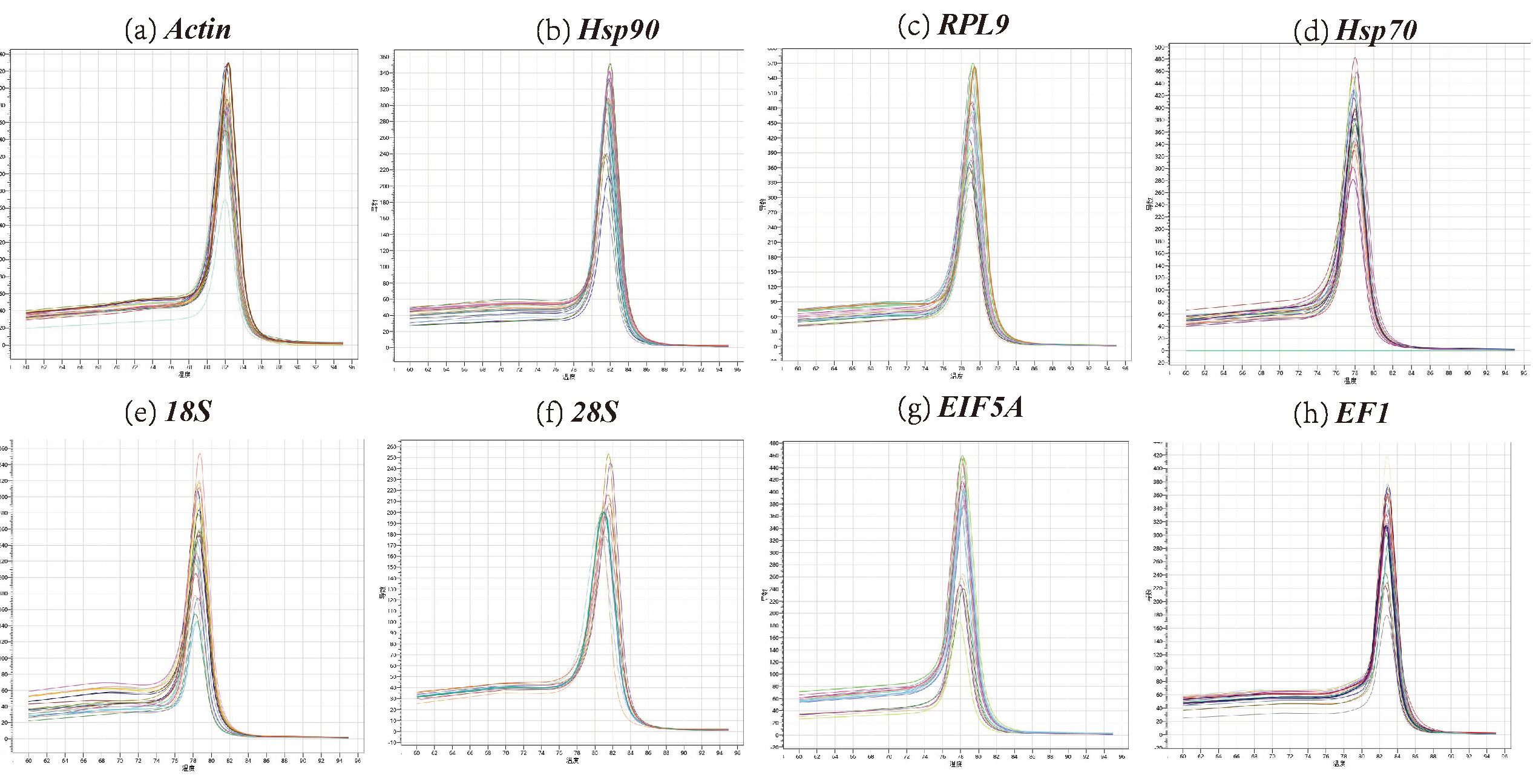

Supplement: Supplementary file 1 — Additional file 1. [file 12864_2023_9843_MOESM1_ESM.zip › Supplementary/FigureS1.tif]

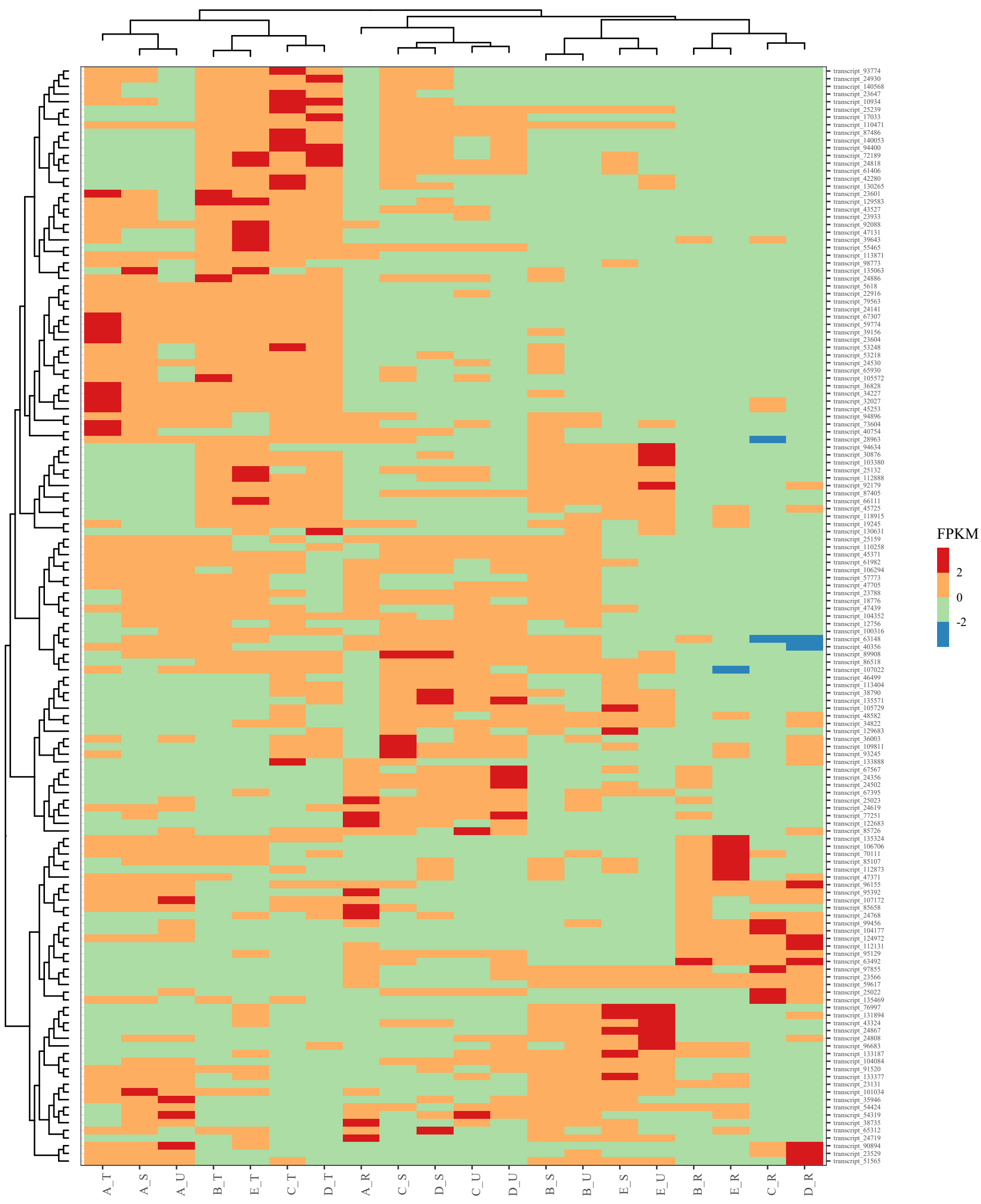

Supplement: Supplementary file 1 — Additional file 1. [file 12864_2023_9843_MOESM1_ESM.zip › Supplementary/FigureS4.pdf]

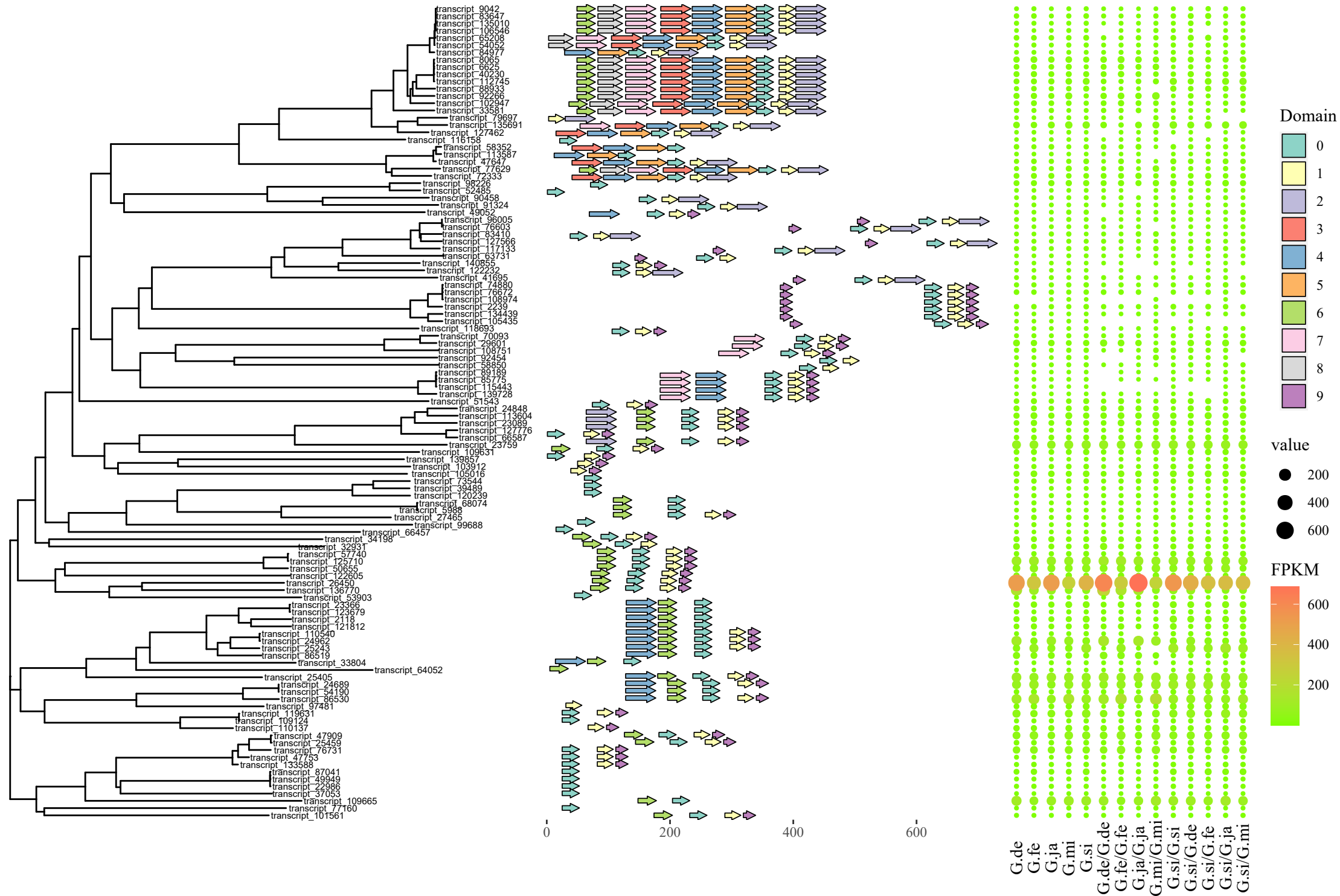

Supplement: Supplementary file 1 — Additional file 1. [file 12864_2023_9843_MOESM1_ESM.zip › Supplementary/FigureS5.pdf]
