# Supplementary material for: Full-length transcriptome characterization and comparative analysis of Gleditsia sinensis
Source: BMC Genomics. 2023 Dec 8;24:757. doi: 10.1186/s12864-023-09843-y (PMC10709882; doi:10.1186/s12864-023-09843-y)
Supplement: Supplementary file 1 — Additional file 1. [file FigureS2.pdf › Supplementary/FigureS2.pdf]

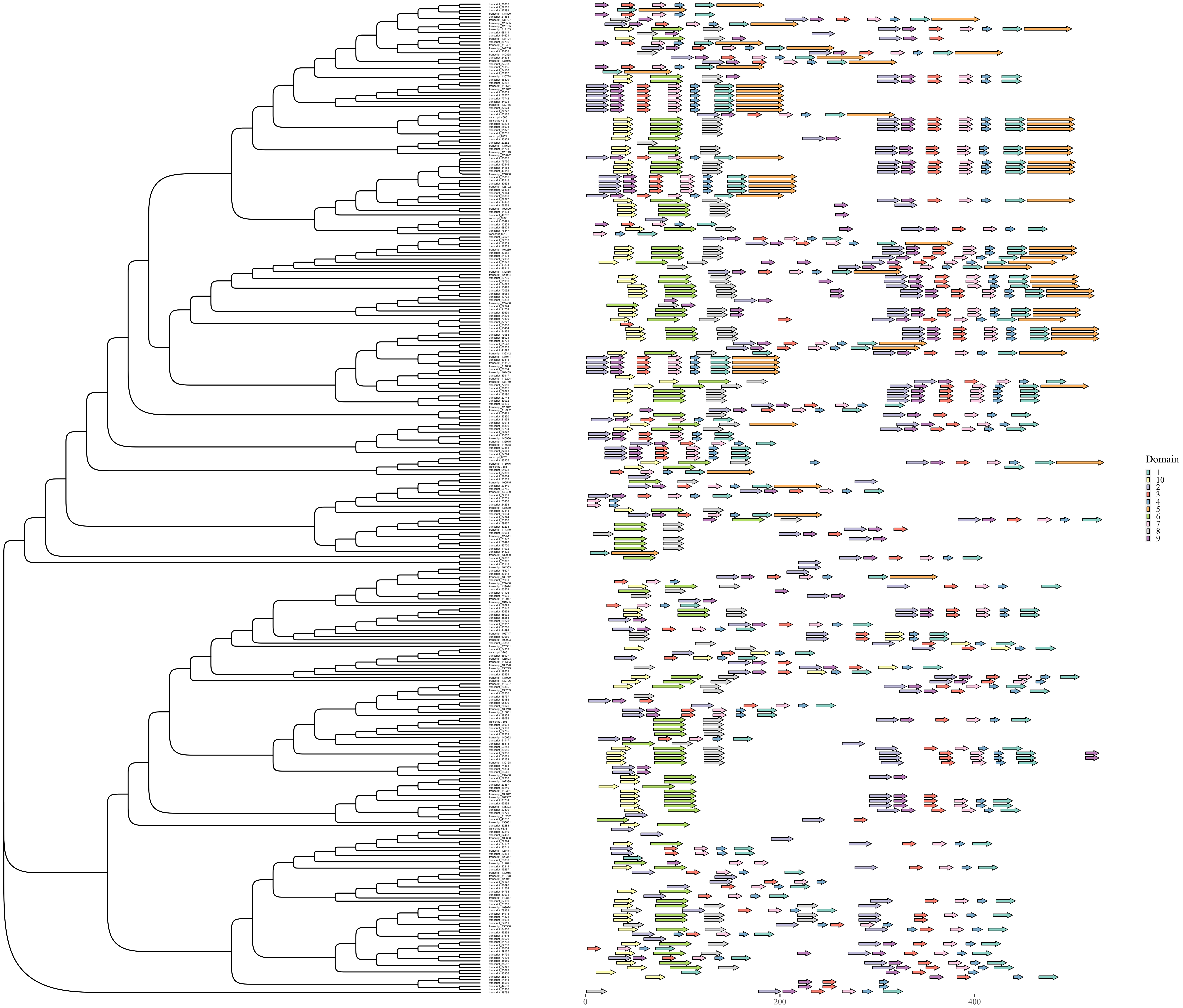
Domain
1
10
2
3
4
5
6
7
8
9
0
200
400
